# Child health vulnerabilities during the COVID-19 pandemic in Brazil and Portugal*

**DOI:** 10.1590/1518-8345.4805.3422

**Published:** 2021-07-02

**Authors:** Ivone Evangelista Cabral, Márcia Pestana-Santos, Lia Leão Ciuffo, Yan do Rosario Nunes, Maria de Lurdes Lopes de Freitas Lomba

**Affiliations:** 1 Universidade Federal do Rio de Janeiro, Escola de Enfermagem Anna Nery, Rio de Janeiro, RJ, Brazil.; 2 Universidade do Estado do Rio de Janeiro, Faculdade de Enfermagem. Rio de Janeiro, RJ. Brazil.; 3 Fellowship holder at the Conselho Nacional de Desenvolvimento Científico e Tecnológico (CNPq), Brazil.; 4 Escola Superior de Enfermagem de Coimbra, Unidade de Investigação em Ciências da Saúde-Enfermagem, Coimbra, Portugal.; 5 Universidade do Porto, Porto, Portugal.; 6 Scholarship holder at the Institutional Program of Scientific Initiation of the Universidade Federal do Rio de Janeiro. Rio de Janeiro, RJ, Brazil.

**Keywords:** Continuity of Patient Care, Comprehensive Health Care, Health Vulnerability, Family Nursing, Primary Care Nursing, Child Health Services, Continuidade da Assistência ao Paciente, Assistência Integral à Saúde, Vulnerabilidade em Saúde, Enfermagem Familiar, Enfermagem de Atenção Primária, Serviços de Saúde da Criança, Continuidad de la Atención al Paciente, Atención Integral de Salud, Vulnerabilidad en Salud, Enfermería de la Familia, Enfermería de Atención Primaria, Servicios de Salud del Niño

## Abstract

**Objective:**

to analyze the vulnerabilities of children in the access to primary health care during the COVID-19 pandemic in Brazil and Portugal.

**Method:**

documentary study based on Brazilian and Portuguese governmental guidelines issued between March and August 2020 regarding access of children to primary health care. Thematic analysis was based on the precepts of health vulnerability.

**Results:**

13 documents were issued in both countries addressing access to vaccination and childcare. Due to the SARS-CoV-2, restrictions were imposed on the circulation of people in social environments, health services, and social protection, decreasing the demand for health services. Both countries continued programs to promote the health of breastfeeding infants. In-person childcare consultations were suspended for low-risk children in both countries. Portugal maintained routine vaccination while Brazil interrupted vaccination in the first 15 days of the pandemic. The countries adopted remote care strategies - telemonitoring, teleconsultation, and mobile applications - to maintain the bond between children and health services.

**Conclusion:**

longitudinality was affected due to restricted access of children to health promotion actions, determining greater programmatic vulnerability. Individual vulnerabilities are related to exposure to preventable and primary health care-sensitive diseases.

## Abstract (pt)

**Objetivo:**

: analisar as vulnerabilidades da criança no acesso aos cuidados na atenção primária durante a pandemia da COVID-19 no Brasil e em Portugal.

**Método:**

pesquisa documental baseada em diretrizes governamentais brasileiras e portuguesas, expedidas entre março e agosto de 2020, sobre o acesso de crianças à atenção primária. A análise temática fundamentou-se nos preceitos da vulnerabilidade em saúde.

**Resultados:**

expediram-se 13 documentos nos dois países sobre acesso à vacinação e à puericultura. A restrição à circulação do SARS-CoV-2 nos ambientes sociais, serviços de saúde e de proteção social reduziu a demanda de atendimento. Mantiveram-se, nos dois países, os programas de promoção da saúde do lactente. O acompanhamento de puericultura presencial, para crianças de baixo risco, foi suspenso nos dois países. Portugal manteve a vacinação rotineira e o Brasil a interrompeu nos primeiros 15 dias da pandemia. Os países adotaram estratégias remotas de atenção - telemonitoramento, teleconsulta e aplicativos móveis - mantendo o vínculo da criança com os serviços de saúde.

**Conclusão:**

a longitudinalidade foi afetada pela redução do acesso à promoção da saúde da criança, determinando maior vulnerabilidade programática. As vulnerabilidades individuais relacionaram-se à exposição a doenças evitáveis e sensíveis à atenção primária.

## Abstract (es)

**Objetivo:**

analizar las vulnerabilidades del niño en el acceso a los cuidados en la atención primaria, durante la pandemia del COVID-19 en Brasil y Portugal.

**Método:**

investigación documental basada en directrices gubernamentales brasileñas y portuguesas, expedidas entre marzo y agosto de 2020, sobre el acceso de niños a la atención primaria. El análisis temático se fundamentó en los preceptos de la vulnerabilidad en la salud.

**Resultados:**

fueron encontrados 13 documentos, expedidos en los dos países, sobre el acceso a la vacunación y puericultura. La restricción de circulación del SARS-CoV-2 - en los ambientes sociales, servicios de salud y de protección social - redujo la demanda de la atención. En los dos países se mantuvieron los programas de promoción de la salud del lactante. El acompañamiento de puericultura presencial, para niños de bajo riesgo, fue suspendido en los dos países. Portugal mantuvo la vacunación de rutina y Brasil la interrumpió en los primeros 15 días de la epidemia. Los países adoptaron estrategias de atención a distancia (telemonitoreo, teleconsulta y aplicativos móviles) manteniendo el vínculo del niño con los servicios de la salud.

**Conclusión:**

la longitudinalidad fue afectada por la reducción del acceso a la promoción de la salud del niño, determinando mayor vulnerabilidad programática. Las vulnerabilidades individuales se relacionaron a la exposición a enfermedades evitables y sensibles a la atención primaria.

## Introduction

The United Nations Children's Fund (UNICEF) released its action agenda in defense of children who are most vulnerable to COVID-19 (Coronavirus disease 2019), caused by the coronavirus (SARS-CoV2), in the context of global health, highlighting the need for urgent actions to avoid a health crisis that results in violating children's rights in the broadest sense^(^ 1 ^)^.

Vulnerability refers to a context of social relations that limits the ability of people to act when institutional support that would ensure social security is no longer available. These are situations that deny people the possibility to effectively exercise their citizenship rights and, consequently, individuals experience insecurity in the present that may frustrate future projects^(^ 2 ^)^.

Even though statistically, children are less likely to be infected with the disease compared to adult and elderly individuals, the COVID-19 pandemic leads to a context of vulnerability. Prevalence of the disease in different countries is below 5% in the child population (from one day to 15 years old), with an even lower number of cases during lactation^(^ 3 ^-^ 4 ^)^. It is unknown why morbidity caused by COVID-19 is less severe in this group though, considering that the virus is transmitted person-to-person when having direct contact with an infected individual or with his/her respiratory droplets. This lower susceptibility has been explained by the lower likelihood of children crowding or traveling to epidemic countries. Even though morbidity and mortality rates are low, one has to be attentive to a greater vulnerability to other diseases not associated with SARS-CoV-2, which may indirectly increase morbidity and mortality in this population.

Another explanation refers to the distribution, maturation, and functioning of viral receptors. SARS-CoV-2 uses the angiotensin-converting enzyme 2 (ACE2) as a cell receptor, which in mice lungs decreases drastically with age. Additionally, it has been found to protect against lung injuries due to infections caused by respiratory viruses and severe acute lung injuries triggered by sepsis, acid aspiration, severe acute respiratory syndrome (SARS), and lethal infection caused by the avian influenza virus. Thus, these findings suggest that children are less susceptible to COVID-19^(^ 5 ^)^.

The duration of the disease (3, 6, and 12 months) was associated, in mathematical modeling applied in hypothetical scenarios, with a restricted supply of child health services, and mortality among 5-year-old children was estimated in 118 countries with low to moderate income levels. The findings show that children are more vulnerable to the effects of the COVID-19 pandemic due to decreased access and coverage of services, among which, vaccination. Decreased access, between 9.8% and 18.5%, in a less severe context (6 months) could cause the death of 253,500 children. In a more severe context (12 months), decreased access between 39.3 and 51.9% could cause more than 1,157,000 deaths. These additional deaths would represent a monthly increase from 9.8 to 44.7% in the number of deaths of children younger than 5 years old^(^ 6 ^)^. The conclusion is that the prolonged effects of the pandemic on children's health could severely harm children and increase the rates of morbidity and mortality due to preventable causes in proportions equal to or greater than that of the COVID-19.

Additionally, conditions affecting children with special health care needs (CSHCN) are more likely to become chronic due to the specificity and differential care required, particularly if follow-up and rehabilitation visits are discontinued^(^ 7 ^)^. In general, vulnerabilities among children imply even greater exposure to mental health diseases such as profound sadness and anxiety, which may worsen with social isolation^(^ 8 ^-^ 11 ^)^.

To address these vulnerabilities, the *Plano Nacional de Saúde Infantil e Juvenil (PNSIJ)* (National Plan for Child and Youth Health) in Portugal, recommended continuing actions intended to promote the growth and development of children, through the *Consulta de Vigilância de Saúde* (Health Surveillance Consultation), as it is a valuable opportunity to screen, assess, intervene and deal with problem situations. PNSIJ reinforces the need to identify children with chronic diseases and/or disabilities and their families and provide continued support, vaccination, early care, and coordinate with the various health care providers^(^ 12 ^)^. Detecting situations that can be corrected early includes monitoring and referral to deal with those situations that interfere in children's health^(^ 12 ^)^.

There has been a dilemma in Brazil in the context of the COVID-19 pandemic. When the health crisis was enacted, in March 2020, the Brazilian Ministry of Health issued a technical note^(^ 13 ^)^, corroborated by the (state and municipal) Health Departments, restricting the care provided to children in primary health care units. The intention was to avoid agglomerations in health units, however, exposed children with greater vulnerabilities to hospitalizations to treat primary care-sensitive conditions (PCSC). Particularly conditions that may result from poor orientation provided by the primary care network to families or from the population's difficulty to understand the broad role of this level of care, centered on the child, family, and community, instead of focusing on the complaint or disease^(^ 14 ^)^.

In this sense, this study's objective was to analyze the vulnerabilities of children regarding access to primary health care during the COVID-19 pandemic in Brazil and Portugal.

## Method

This is a documentary study in which data sources were regulatory devices addressing childcare in the context of the COVID-19 pandemic. This type of research is taken as the object of investigation, that is, sources of information that clarify questions and serve as evidence for other new questions, the content of which has not been previously analyzed. Thus, these are primary sources used to answer the study objectives^(^ 15 ^)^.

Documentary research begins with finding relevant, representative, and credible documents that record the investigated facts. It occurs in three stages: preliminary assessment, documentary analysis, and interpretation. The first stage consists of seeking the dimensions of the context in the texts, the authors, authenticity and reliability, nature, key expressions, and logic of the texts. In documentary analysis, thematic or lexical signifiers are extracted from texts, seeking the simplest elements. Then, words or ideas are synthesized into similar record units, which are coded into thematic units to form themes. At this point, the researcher may choose the method of analysis. Interpretation or inference rests on the theoretical framework to apprehend meaningful elements of new knowledge.

Using government regulations issued or in force in the context of the COVID-19 pandemic was the criterion used in the first stage. The documentary analysis framework (Figure 1) includes ordinances (from governmental and non-governmental organizations), technical notes, protocols, and guidelines.

**Figure 1:** Documents guiding childcare in primary health care during the COVID-19 pandemic. Brazil and Portugal, 2020

BRAZIL/ DOCUMENT CODE	PORTUGAL/ DOCUMENT CODE
1. (Code)-Ordinance MS-356, from 11/03/2020. Ordinance Ministry of Health (MS) No. 356, from March 11th, 2020. Available from: http://www.in.gov.br/en/web/dou/-/portaria-n-356-de-11-de-marco-de-2020-247538346	1. (Code)-DL2A-2020, de 20/03/2020. Decree-Law (DL) 2A/2020 from March 20th, 2020. Available from: https://dre.pt/web/guest/pesquisa/-/search/130473161/details/normal?p_p_auth=e8JSo0VO
2. (Code)-Ordinance MS-430, from 19/03/2020. Ordinance Ministry of Health (MS) No. 430, from March 19th, 2020. Available from: http://www.in.gov.br/en/web/dou/-/portaria-n-430-de-19-de-marco-de-2020-249027837	2. (Code)-D-3331-E, de 15/03/2020. Order (D) No. 3301-E/2020. Diário da República No. 52-B/2020, 2nd Supplement, Series II from 2020-03-15. Available from: https://dre.pt/web/guest/home/-/dre/130277343/details/maximized?serie=II&day=2020-03-15&date=2020-03-01&dreId=130277339
3. (Code)-Ordinance MS-454, de 20/03/2020. Ordinance Ministry of Health (MS) No. 454, from March 20th, 2020. Available from: http://www.in.gov.br/en/web/dou/-/portaria-n-454-de-20-de-marco-de-2020-249091587	3. (Code)-DGS - 160_80_v1 from March 25th, 2020. Directorate-General for Health (DGS). Communication 160_80_v1 de 25/03/2020 - Available from: http//www.dgs.pt/a-direccao-geral-da-saude/comunicados-e-despachos-do-director-geral/cumprimento-do-programa-nacional-de-vacinacao-durante-a-epidemia-de-COVID-19-medidas-de-excecao-pdf.aspx.Lisboa: 2020.
4. (Code)-NT-MS_23/03/2020. Technical note (TN) from the Ministry of Health (MS), from March 23rd, 2020. Annex.	4. (Code)-DGS_008/2020, from March 26th, 2020. Directorate-General for Health (DGS). INFORMATION - Programa Nacional de Saúde Infantil e Juvenil. Available from https://www.dgs.pt/directrizes-da-dgs/normas-e-circulares-normativas/norma-n-0102013-de-31052013-jpg.aspx. Lisbon: 2020
5. (Code)-CONASS. 04/2020. CONASS. March/2020. National Council of Health Secretaries (CONASS). March/2020. Available from: http://www.conass.org.br/wp-content/uploads/2020/04/ATENDIMENTO-DA-REDE-DE-ATENCAO-A-SAUDE-PANDEMIA.pdf	5. (Code)-DGS-004/2020 from March 23rd, 2020. Directorate-General for Health (DGS). Norm - 004/2020 de 23/03/2020, updated on April 25th, 2020. Available from: https://www.dgs.pt/directrizes-da-dgs/normas-e-circulares-normativas/norma-n-0042020-de-23032020-pdf.aspx. Lisbon: 2020.
6. (Code)-Protocol COVID-19_APS_2020. Brasil. Ministry of Health. Secretariat of Primary Health Care. Available from: https://pesquisa.bvsalud.org/portal/resource/pt/biblio-1087335	6. (Code)-DGS_026/2020, from May 19th, 2020. Directorate-General for Health (DGS). Orientation - 026/2020 from May 19th, 2020. Available from https://www.dgs.pt/directrizes-da-dgs/orientacoes-e-circulares-informativas/orientacao-n-0262020-de-19052020-pdf.aspx
7. (Code)-CONASEMS/ CONASS. 08/2020. National Council of Municipal Health Departments. - CONASEMS. National Council of Health Secretaries- CONASS. COVID-19 Guia Orientador para o enfrentamento da pandemia na Rede de Atenção à Saúde. [Guide to deal with the pandemic in the Primary Health Network] 2nd edition. Brasília (DF): August 2020. Available from: https://www.conass.org.br/guia-orienta-estados-e-municipios-para-o-enfrentamento-da-pandemia-de-covid-19-na-rede-de-atencao-a-saude/	

Geographically, Brazil and Portugal were chosen to be the study setting because both countries have universal health systems and a large service network in which nursing workers provide care to children in the primary health care context. As for the particularities of the two countries concerning how the delivery of care is organized to respond to different territorial and demographic dimensions, public financing of the health system establishes a favorable environment for implementing the guidelines of the World Health Organization to deal with the pandemic.

Both health systems are based on the notion that health is a fundamental right determined by the constitution and based on organic laws that regulate childcare with absolute priority, considering the vulnerability of this population. The Ministry of Health has the responsibility to formulate policies and national health plans to promote health, prevent diseases, reduce harm, and promote rehabilitation. Management models - centralized in Portugal, decentralized in Brazil - put into operation strategies and actions to meet the population's health needs and governance needs in the territory, even in underfunding contexts of both systems^(^ 16 ^-^ 19 ^)^.

Regulatory documents issued from March to August 2020 were selected, which corresponds to the period in which the services were organized to meet the needs of patients within the COVID-19 pandemic.

Key expressions were extracted during the preliminary and exhaustive reading of the documents (Figure 1) and were used to answer analytical questions (Figure 2) regarding programmatic guidelines concerning the health promotion and harm reduction within child health in primary health care.

**Figure 2:** Questions and key expressions extracted from the documents' units of analysis. Brazil and Portugal, August 2020

QUESTIONS During the COVID-19 pandemic, in primary health care...	KEY EXPRESSIONS
What were the guidelines concerning the promotion of child health?	Newborns Neonatal screening Breastfeeding, breastfeeding in the primary health care unit, in the maternity Growth and development surveillance Vaccination Children Service/follow-up/consultations: scheduled, non-priority, spontaneous, suspended Child immunization/vaccination; expanded office hours, service reorganized/rearranged, priority/priori Exclusive breastfeeding, child feeding: in the primary health care unit; at home
What were the strategies recommended to promote child health?	Breastfeeding: maintenance, interruption, express milk Financial support Remote strategies: telehealth/telemedicine telemonitoring/teleconsultation, video consultation, applications In-person/face-to-face strategies: home visits
What were the strategies recommended to monitor children with chronic diseases and clinical management of COVID-19 in childhood?	Flu syndrome, acute respiratory distress syndrome/ARDS, COVID-19/SARS-CoV-2 Hospitalization/referral/reference Vigilance: actions Common conditions: reception; access; management; services; actions

Regulatory documents in the public domain are sources of information that do not require approval by an Institutional Review Board (CEP/CONEP - Resolution No. 510, from April 7th, 2016, Brazilian Council of Health, article 1st, sole paragraph, item III).

In the documentary analysis, the answers to the questions were organized into four analytical tables. Each contained the documental source and the key expressions extracted from the tests that compose the units of record (UR). Then, the URs were brought together by convergent lexical signifiers to form thematic units, which were regrouped into themes according to common and specific meanings, based on the thematic analysis guidelines. In this type of thematic analysis, the relationships between the concepts extracted from data are determined, comparing them with different situations to generate possibilities to interpret content in the light of the theoretical framework^(^ 20 ^)^.

Based on these guidelines, the coding of the convergent thematic units of each country was regrouped in new tables containing common and different concepts, which were then interpreted in the light of the theoretical framework of vulnerability^(^ 2 ^,^ 21 ^)^.

Individuals become resilient when experiencing socially vulnerable situations because they persist, assert themselves as individuals, and recognize fragility as a condition inherent to living beings. The specificity of resilience is defined in the limits that life circumstances allow as part of a creative or innovative path^(^ 2 ^)^.

The concept of vulnerability in the health field, from the perspective of human rights, has two collective dimensions (social and programmatic) and one individual dimension^(^ 21 ^)^.

Taking the social collective dimension^(^ 21 ^)^ as a reference in the child health field, social standards and familial cultural references are applied. The wellbeing of a child results from the family having access to employment and income; from defending the child's best interest; and having access to conditions that ensure fundamental rights (life, health, education, culture, leisure, sports, etc.). It also includes access to information, the government committing to life, and the family's and the child's social wellbeing.

Within the programmatic collective dimension, governments have the responsibility to meet the health needs of people, by establishing public policies that protect the children's best interests. In this sense, there has to be planning and assessment of care, the supply of material, and human resources that favor the implementation and maintenance of policies, in addition to ensuring governance, political, institutional, and material sustainability, connections, and intersectoral activities. The organization of the health sector should ensure access and quality of services, with multidisciplinary teams working with an interdisciplinary approach to preventive measures and integration of care. It also includes the sensitization of workers regarding human rights, planning, and assessment of health services^(^ 21 ^)^. The individual dimension includes values, interests, beliefs, desires, knowledge, attitudes, behavior, social networks, friendships, marital and family relations, mental health, and physical constitution^(^ 21 ^)^.

The interpretation of data indicated two main themes: programmatic vulnerabilities concerning preventable diseases, flu syndrome, and COVID-19 in primary health care, and strategies to deal with COVID-19-related vulnerabilities.

## Results

The 13 documents selected presented guidelines concerning the care provided to children during the COVID-19 pandemic: seven of which were from Brazil and six from Portugal. At the beginning of the pandemic, both countries regulated and established a national plan to deal with it, applying the guidelines provided by the World Health Organization regarding social distancing measures to decrease or interrupt the flow of people in public spaces (schools, health services, and daycare services), restricting the provision of services to urgent situations.

At the same time, these measures established a sanitary cordon to prevent the spread of the SARS-CoV-2 and contain the COVID-19, it exposed children to illnesses due to other preventable causes and worsened the chronic nature of pre-existing conditions.

### Programmatic vulnerabilities concerning preventable diseases, flu syndrome, and COVID-19 in primary health care

Both countries developed programmatic guidelines during the COVID-19 pandemic for primary health care intended to ensure health promotion and harm reduction in the context of child health. In Brazil, routine vaccination, which is a health promotion strategy, was postponed for three weeks in the entire national territory, as a measure to decrease contacts.

*Parents and/or legal guardians of children shall not seek vaccination services in the 1st phase (March 23rd to April 15th) of the influenza vaccination campaign. The purpose is to decrease contact with elderly individuals, a group at the highest risk of experiencing complications due to COVID-19, and decrease contact with children who are asymptomatic carriers and disseminators of respiratory diseases* (Brasil-NT-MS_23/03/2020 p.1 and Annex). Exceptionally, children with *delayed vaccination: who sought the service for not knowing vaccination was postponed and need to update the immunization scheme, will be vaccinated to take advantage of the opportunity* (Brasil-NT-MS_23/03/2020 p.1 and Annex).

Each state of the Brazilian federation adapted its vaccination schedule to meet local epidemiological specificities. The National Council of Health Departments initially recommended that state departments expanded *the H1N1 coverage according to priority groups and the vaccination schedule established by the Ministry of Health* (Brasil. CONASS. 04/2020). In August 2020, the *Guia Orientador para o enfrentamento da pandemia na Rede de Atenção a Saúde* [Instruction Guide to deal with the pandemic in the Health Care Network] (Brasil. CONASEMS/ CONASS. 08/2020), of the (state and municipal) Councils of Health Secretaries included guidelines for primary health care, highlighting that *primary health care units* should ensure the *continuity of vaccination*.

In Portugal, routine vaccination services remained active. There was low demand from parents though, out of fear of contamination by the SARS-CoV-2. For this reason, the Directorate General of Health had to inform the population and health workers about the continuation of vaccination, recommending the update of delayed vaccination schemes. In this sense, all the vaccines on the mandatory immunization schedule in Portugal remained available to the families, as part of health promotion actions, especially those that determined greater morbidity and mortality.

*(...) inform that vaccination should not be postponed (...) to comply with the Programa Nacional de Vacinação [National Vaccination Program]. ...immunization recommended in the first year of life, which confers early protection against 11 diseases (...). At 12 months of age, meningococcal-C vaccine, (...) measles, mumps, and rubella. (...) Children with these vaccines overdue are recommended to be vaccinated as soon as possible*. (Portugal-DGS_008/2020-26/03/2020, p. 1)

In the epidemic phase of COVID19, CONASS sought to organize and standardize the coordination of primary health care. In this sense, it recommended the management and control of health conditions through Healthcare Networks. The states are oriented to maintain the monitoring of child growth and development, neonatal screening, and childcare nursing consultation in the health unit or at home. *Childcare consultations for high-risk children, the collection of neonatal screening between the 3rd and 5th day of life among newborns: "5th-day actions", preferably at home or scheduled at intervals; Nurse consultation focusing on breastfeeding and weight gain* (Brasil-CONASS. 04/2020). One month later, *child health actions* remained the same, except for the coverage of childcare consultations, that is, which included *medium-risk children with fragile care provided by the family (...) focusing on the development and nutritional monitoring* (Brasil. CONASEMS/ CONASS. 08/2020).

In Portugal, the recommendations also involved not interrupting neonatal surveillance and the *procedures provided in the National Neonatal Screening Program (Heel-Stick Test).* These tests shall be *performed between the 3rd and 6th day after birth, in the maternity hospital or on the 1st medical appointment (...) when eligibility criteria for BCG vaccination are met* (Portugal-DGS_008/2020-26/03/2020, p. 2)

Both Brazil and Portugal adopted the same recommendations to maintain breastfeeding, determined at a central level. In case of infection, a woman's desire and her clinical conditions would determine whether to maintain breastfeeding, recommending manually expressing breast milk implementing hygiene measures.

*Breastfeeding may continue among mothers infected with the virus according to the Ministry of Health's COVID19 Protocol. If the lactating woman is in the acute phase of the disease and the staff is unsure whether direct contact should be allowed, breast milk can be expressed and offered to the newborn (...) Washing hands before touching the baby at the time of feeding; wearing a face mask during breastfeeding* (Brasil-CONASS. 04/2020; Brasil. CONASEMS/ CONASS. 08/2020).

*Infected mothers shall wear a mask to breastfeed, after hand and breast hygiene. (...) Wear a mask to mechanically express milk after hand and breast hygiene. Breast milk can be offered to the NB by a healthy caregiver.* (Portugal-DGS_026/2020_19/05/2020, p. 8).

In Portugal, routine consultations during the health crisis were scheduled to coincide with the vaccination days according to the national immunization schedule. The remaining surveillance consultations were suspended. Through teleconsultation, health workers (physicians or nurses) assessed the need to refer children to in-person consultations. Recommendation to attend at least six consultations in the first year of life remained.

*Despite the exceptional situation, because of the vulnerable condition of children, surveillance consultations in the 1st year of life, including a visit at 12 months of age, shall not be postponed (...) children who delay return visits shall be called immediately. One consultation between 18 and 24 months of age and another at the age of 5 years old.* (Portugal-DGS_008/2020_26/03/2020, p. 2).

Among children, the ones most vulnerable to the flu syndrome and COVID-19 are those with special health needs (CSHCN) who present a greater risk of complications.

*Risk conditions for complications. Children <5 years old (while the greatest risk of hospitalization is among those younger than 2 years old, especially younger than 6 months old, with a higher mortality rate) (...) who present lung diseases, cardio-vasculopathies, kidney diseases, liver diseases, hematological diseases, metabolic disorders, or neurological and developmental disorders that can compromise respiratory function or increase the risk of aspiration, immunosuppression associated with medications, neoplasms, HIV/AIDS or obesity.* (Brasil-Protocolo COVID-19_APS_2020, p. 17).

Children with comorbidities present more individual vulnerabilities to the flu syndrome and COVID-19, indicating the need for further assessment in a referral center or specialized care center. For the nurses working in primary health care units, these vulnerabilities require surveillance for the following signs and symptoms and symptoms that indicate severe flu syndrome:

*Respiratory deficit (...) pulse oximetry saturation <95% in room air; deficit in the cardiovascular system; Additional warning signs and symptoms such as lack of appetite for breastfeeding or fluid intake; clinical worsening of underlying diseases; alteration in mental status, confusion, lethargy, or convulsions.* (Brasil-Protocolo COVID-19_APS_2020, p. 13).

The Brazilian Ministry of Health provided financial support to the states' and cities' health departments to fight COVID-19 and manage the more common health conditions within primary health care, to expand working hours, access to control measures, actions, and services.

### Strategies to deal with COVID-19-related vulnerabilities.

*(...) for assistance, diagnosis, treatment, prevention, outbreak control, and interruption of the COVID-19 transmission chain;(...) access to essential actions and services provided by the Family Health teams and Primary Health Care teams to manage common health conditions and provide clinical actions and services and health surveillance.* (Brasil-Portaria-MS-430_19/03/2020, art 2º).

Family health or primary health care units should commit to maintaining health workers in all work shifts, *to ensure medical and nursing consultations, throughout working hours of health units, 60 or 75 hours per week.* (Brasil-Portaria-MS-430_ 19/03/2020, art 2º/artº 4º).

In Portugal, except for vaccination and to prevent an overload of health services, the flow of ill individuals was exceptionally regulated and decreased. The *implementation of these measures on the part of health institutions was intended to postpone non-urgent services.* (Portugal-DGS_Comunicado_160_80_v1-25/03/2020, p. 1). At the same time, the services' capacity was adjusted to meet the demand of patients with COVID-19. The hiring of health workers *for a four-month period (...)* was authorized *to reinforce (...) prevention, containment, mitigation, and treatment of COVID-19*. (Portugal-D-3331-E-15/03/2020).

Personal and environmental hygiene is recommended for primary health care services to manage the flu syndrome, in addition to clinical-therapeutic management with pharmacological measures to relieve respiratory discomfort and pain. Premature children with chronic lung disease should be referred to a specialized care center. The National Council of Health Secretaries recommended strategies to keep the care provided to children in primary health care and specialized networks. *High- and medium-risk children shall be monitored in person or through telephone or WhatsApp (...).* (Brasil-CONASS. 04/2020; Brasil. CONASEMS/ CONASS. 08/2020).

Specific strategies were established in Portugal for the pediatric population due to its greater vulnerability, *maintaining and monitoring the health of the child population during this period of exception.* (Portugal-DGS_008/2020_26/03/2020, p.1).

The family teams of each health care service should *update the contact of caregivers, keeping the activity that involves identifying children with specific needs, at a risk situation, or with greater vulnerability in the areas under coverage.* (DGS_008/2020_26/03/2020, p. 1). The teams were also warned to pay *special attention, and implement measures and keeping monitoring health needs accruing from children's specific situations*. (Portugal-DGS_008/2020_26/03/2020, p. 1).

Criteria for monitoring and surveilling infected children, hospitalized and at home, favored differentiated screening according to the children's care needs. Some of the *(...) Criteria for habitability and feasibility of shelter-at-home include having an accessible landline or mobile phone (...).* (Portugal-DGS_004/2020, de 23/03/2020, p. 18).

In summary, the health systems ensured the continuity of care for children with limited access within primary health care (Figure 3).

**Figure 3:** Synthesis of child protection strategies in the context of the COVID-19 pandemic. Brazil and Portugal, August 2020

Strategies		
Intervention areas	Brazil	Portugal
Vaccination	Temporarily suspended Resuming on April 16th, 2020. Working hours were expanded. Separate service entrance door for the children.	Maintained
Growth and development surveillance	Routine consultations suspended. Low- and medium-risk children recommended for teleconsultation.	Maintained on vaccination days
Neonatal screening	Maintained through collection at home.	Collection remained in the health unit.
Maintenance of breastfeeding even if the nursing mother had the COVID-19 Use and replacement of mask whenever it is humid Hand washing for 20 seconds before breastfeeding.	Maintained in both countries	
Outpatient monitoring of children with special and differentiated health needs (CSHCN)	In-person visits suspended	In-person or telemonitoring (application of telephone)
Monitoring of children infected by SARS-CoV-2	Both countries: telemonitoring (applications, telephone) Home care (separate beds, masks, individual bathrooms, environment, utensils, and hands hygiene). Hospitalization in cases of severe respiratory distress syndrome/COVID-19	

## Discussion

In Brazil, on the first day of the health crisis caused by the COVID-19 pandemic, numerous children stopped being vaccinated in a period of increased measles outbreak.

In Portugal, vaccination continued according to the schedule provided by the national immunization program though many families failed to take their children to vaccination for fear of contamination. Government agencies rapidly broadcast alerts in all Portuguese open TV channels, emphasizing the importance of adhering to the immunization schedule due to the risk of recurrence of severe childhood illnesses, such as measles. Each health service was also responsible for calling up children with scheduled vaccinations or already behind the vaccination schedule^(^ 22 ^)^.

Vaccination is an important health promotion strategy that can reduce diseases caused by preventable causes and hospitalizations due to primary health care-sensitive conditions that may coexist with other severe acute respiratory syndromes, such as the case of COVID-19^(^ 23 ^)^. In Africa, in a high-impact context of the COVID-19 pandemic, a simulated analysis based on a mathematical model estimated that, if routine immunization was kept, 84 deaths would be avoided among children for each death caused by the SARS-CoV-2 infection acquired during routine vaccinations. The risk-benefit ratio for vaccinated children is 85,000; for their siblings (<20 years old), 75,000; their parents or caregivers (between 20 and 60 years old), 769; and elderly individuals, 96. In a low-impact scenario, health benefits are close to deaths avoided when there are measles outbreaks. The risk-benefit ratio for the families of vaccinated children is 3 (0-10), but if only the risk for vaccinated children is considered, the risk-benefit relationship is 3,000. Thus, routine childhood vaccination is recommended even in the face of logistic restrictions, staff shortages, or reallocation of financial resources during the COVID-19 pandemic^(^ 24 ^)^.

Preliminary evidence suggests that even though children are as susceptible as adults to be infected by the SARS-CoV-2, they are less likely to show symptoms or manifest the most severe forms of the disease. The role of children in the spread of the virus remains uncertain. Gastrointestinal symptoms are more common than respiratory symptoms among children, while most children with SARS-CoV-2 experience fever, have one documented contact, generally showing symptoms before them^(^ 25 ^)^.

In this sense, when there are barriers to access, whether these are caused by temporary governmental guidelines or the families' fear that their children will be exposed to viral load, there is a risk of increasing vulnerabilities of children to preventable diseases and hospitalizations due to primary health care-sensitive conditions^(^ 5 ^)^. In April 2020, UNICEF warned about the risk of more than 117 million children from 37 countries not being vaccinated against measles due to the COVID-19 outbreak^(^ 26 ^)^. The World Health Organization and the Pan-American Health Organization issued guiding principles and immunization programs in the context of the pandemic, warning about the risk of preventable morbidities and mortality to increase in the context of the COVID-19 pandemic^(^ 26 ^-^ 27 ^)^.

In Brazil, growth and development surveillance was interrupted as well as in-person childcare consultations. Priority was given to maintaining newborn care, particularly vaccination in the maternity, breastfeeding protection and promotion, and neonatal screening for children over the age of 30 days. Routine vaccination was resumed after three weeks.

The programmatic guidelines of health promotion and harm reduction in Portugal focused on neonatal screening and follow-up consultations on the days that coincided with the immunization program. This synchronous scheduling saved visits to primary health care services and teleconsultation was expanded during the COVID-19 pandemic.

As for childcare, both countries presented programmatic vulnerabilities that were associated with restricted face-to-face access to primary health care intended to decrease the spread of the COVID-19. Consequently, this measure affected the longitudinality of follow-up during the pandemic with no expectation when it will end, whether due to a decision on the part of the parents (social isolation) or government programmatic guidelines.

Due to individual vulnerability and clinical fragility, CSHCN received special care in both countries to detect and monitor flu syndrome due to the SARS-CoV-2. To prevent the most severe manifestations of the COVID-19, sheltering-in-place for 14 days is recommended, along with telephone review monitoring every 24 h; face-to-face home consultations; resting; balanced diet; and a good supply of fluids. A monitoring interval of 48 h is recommended for the remaining children with flu syndrome along with the remaining measures recommended to all groups of children.

Recommendations regarding primary health care involve warning family health teams within the primary health care to incorporate follow-up within telehealth, especially among children whose social life exposes them to a greater vulnerability that affects the resoluteness of flu syndrome^(^ 28 ^)^.

To monitor flu syndrome and COVID-19, programs and activities were implemented together with other entities and community organizations. In this sense, the Brazilian Ministry of Health provided financial support to (state and municipal) Health Departments to ensure that working hours would be extended for medical and nursing consultations and the unit's physical space would be rearranged. One of the measures to fight the COVID-19 was to implement alternatives to avoid agglomerations and intense circulation of people in the services and preventing children from having contact with more vulnerable population groups, for instance.

In Portugal, the National Health Service had been computerized before COVID-19, which facilitated inter-institutional cooperation (Primary Health Care, Hospitals, Community Pharmacies), maximizing the efficiency of the Health System in an integrated and joint response. The existence and reinforcement of remote care services, as well as the possibility to have access to the prescription of medications of continuous use without the need to commute to health services, ensured health surveillance via teleconsultation.

The Portuguese context differs from the Brazilian context to the extent that the remote care system was created in Brazil only to monitor the flu syndrome and COVID-19. The use of teleconsultation and telehealth to manage the flu syndrome and COVID-19, childcare of low-risk children, were recommended by CONASS/CONASEMS, though there were restrictions in most Brazilian cities; reduced streaming quality compromised the transmission of data in this period. Limited access to the Internet for most of the Brazilian population^(^ 29 ^)^, when compared to the Portuguese population^(^ 30 ^)^, increases the programmatic vulnerability of children in the context of the COVID-19 pandemic.

Documentary analysis in spaces of public transparency of governmental bodies may be a limitation to further analysis. Methodologically, documentary research restricts the analytic focus to the content of the documents accessed, without listening to the services' managers or users.

As for the implications for the advancement of knowledge, we emphasize the institutionalization of telehealth and the rearrangement of services to resume childcare consultations. Home visits are necessary in the territory covered to identify those children missing vaccination and registering children who were born during the pandemic. Successful experiences within telehealth^(^ 31 ^)^, in other conditions, may reduce harm related to individual and social vulnerabilities accruing from social isolation.

The implementation of the national system of telemonitoring is a lesson learned from the Portuguese health system that may favor universal access of the child population to health services. In Brazil, limited access to the Internet on the part of families with economic restrictions increases the individual vulnerabilities of children, particularly infants, young children, CSHCN with disabilities, chronic diseases, or other comorbidities. The non-availability of in-person consultations in primary health care units may increase the risk of exposing these children to complications of the flu syndrome to severe acute respiratory syndrome (SARS). Consequently, greater agility is needed to access the specialized care network, having primary care as coordinator of care, in health systems based on social wellbeing^(^ 32 ^)^.

## Conclusion

In Portugal, health institutions organized the care provided to children who presented a respiratory condition without interrupting the immunization program. Measures specific to the Portuguese context were scheduling childcare consultations that coincided with the vaccine days and expanding nurses' and physicians' use of the teleconsultation. In Brazil, vaccination was temporarily suspended for three weeks to reorganize the care provided to individuals with COVID-19 or flu syndrome. Additional measures were the partial suspension of childcare consultations, the extension of health services' working hours, and teleconsultation to monitor flu syndrome and COVID-19.

Both countries ensured universal access, longitudinality, integrality, decentralized management, and social participation. Measures to contain the spread of the SARS-CoV-2 were implemented, addressing cases, maintaining neonatal screening, and hiring more health workers. There were, however, limitations imposed on the integral delivery of care due to the restrictions on in-person consultations provided within the primary health care scope and inputs to treat preventable diseases and diseases not associated with COVID-19. These measures influenced programmatic, social, and individual vulnerabilities. Social vulnerabilities accrued from social isolation, a strategy implemented to deal with the health crisis caused by the COVID-19 worldwide.

On the other hand, incentives and guidelines to expand coverage and access of children with flu syndrome and COVID-19 to health services affected the longitudinality of health promotion actions. Additionally, it increased the vulnerability of children with special needs and more intensively exposed to diseases and primary health care-sensitive hospitalizations. During the duration of social isolation and distancing measures, the low supply of health services may affect access and longitudinality of health promotion actions, particularly due to decreased immunization coverage and growth and development surveillance.
